# Enhanced Antibacterial Ability of Electrospun PCL Scaffolds Incorporating ZnO Nanowires

**DOI:** 10.3390/ijms241914420

**Published:** 2023-09-22

**Authors:** Jingjing Tian, Thomas E. Paterson, Jingjia Zhang, Yingxing Li, Han Ouyang, Ilida Ortega Asencio, Paul V. Hatton, Yu Zhao, Zhou Li

**Affiliations:** 1 Medical Science Research Center, Peking Union Medical College Hospital, Chinese Academy of Medical Sciences and Peking Union Medical College, Beijing 100730, China; tianjing311@163.com (J.T.); chnxxlyx@126.com (Y.L.); 2 School of Clinical Dentistry, University of Sheffield, Sheffield S10 2TA, UK; t.paterson@sheffield.ac.uk (T.E.P.); i.ortega@sheffield.ac.uk (I.O.A.); paul.hatton@sheffield.ac.uk (P.V.H.); 3 Department of Clinical Laboratory, Peking Union Medical College Hospital, Chinese Academy of Medical Sciences and Peking Union Medical College, Beijing 100730, China; zczjj2009@126.com; 4 School of Nanoscience and Engineering, University of Chinese Academy of Sciences, Beijing 101408, China; ouyanghan@ucas.ac.cn; 5 Department of Orthopaedic Surgery, Peking Union Medical College Hospital, Chinese Academy of Medical Sciences and Peking Union Medical College, Beijing 100730, China; 6 Beijing Key Laboratory of Micro-Nano Energy and Sensor, Beijing Institute of Nanoenergy and Nanosystems, Chinese Academy of Sciences, Beijing 101400, China

**Keywords:** electrospinning, ZnO nanowire, antimicrobial, PCL scaffold, tissue engineering

## Abstract

The infection of implanted biomaterial scaffolds presents a major challenge. Existing therapeutic solutions, such as antibiotic treatment and silver nanoparticle-containing scaffolds are becoming increasingly impractical because of the growth of antibiotic resistance and the toxicity of silver nanoparticles. We present here a novel concept to overcome these limitations, an electrospun polycaprolactone (PCL) scaffold functionalised with zinc oxide nanowires (ZnO NWs). This study assessed the antibacterial capabilities and biocompatibility of PCL/ZnO scaffolds. The fabricated scaffolds were characterised by SEM and EDX, which showed that the ZnO NWs were successfully incorporated and distributed in the electrospun PCL scaffolds. The antibacterial properties were investigated by co-culturing PCL/ZnO scaffolds with *Staphylococcus aureus*. Bacterial colonisation was reduced to 51.3% compared to a PCL-only scaffold. The biocompatibility of the PCL/ZnO scaffolds was assessed by culturing them with HaCaT cells. The PCL scaffolds exhibited no changes in cell metabolic activity with the addition of the ZnO nanowires. The antibacterial and biocompatibility properties make PCL/ZnO a good choice for implanted scaffolds, and this work lays a foundation for ZnO NWs-infused PCL scaffolds in the potential clinical application of tissue engineering.

## 1. Introduction

Polycaprolactone (PCL) scaffolds are widely used in tissue engineering applications for bone, cartilage, skin, dental and nerve tissues because of their biodegradability and ease of moulding into different forms [1]. However, infections after implantation of PCL scaffolds caused by a lack of effective antibacterial ability are currently a common complication [2]. Hence, to improve their anti-infection capacity, combinations of PCL scaffolds with many other materials and drugs have been explored. Many silver (Ag) and Ag nanomaterials have been incorporated into PCL scaffolds for anti-infection activity as tissue engineering scaffolds [3,4] because Ag exhibits broad-spectrum activity against various bacteria, including antibiotic-resistant bacteria, and low toxicity to mammalian cells [5]. Additionally, PCL/PLA fibres containing Ag nanoparticles have also been reported to improve antibacterial activity [6]. However, the potential toxicity of Ag nanoparticles in the skin, kidney, respiratory system, hepatobiliary system, immune system and reproductive system cannot be ignored [7], which limits the wide clinical applications of Ag-containing PCL scaffolds.

ZnO is recognized as a bio-safe material and its use in cosmetic products is approved by the Food and Drug Administration (FDA) [8]. In addition, ZnO nanostructures have been shown to be biodegradable and biocompatible [9,10] and can promote the adhesion, growth and differentiation of several cell lines owing to the presence of OH groups on the surface [11,12], making them an extremely popular material in biomedical applications. ZnO nanostructures such as nanoparticles and nanorods also have been incorporated into many polymers and bioactive ceramics, such as PCL and PLGA, to improve mechanical and antimicrobial properties [13,14,15,16]. ZnO NW modified textile and its application in biosensing, photocatalysis and antibacterial was also investigated [17]. However, there have been few reports about the incorporation of ZnO NWs into polymers or bioactive ceramics. More importantly, we have researched the ZnO NW modified carbon cloth and its promising antibacterial property. ZnO NWs have the advantages of easy synthesis and are easy to combine with other nanoparticles based on our previous investigations [18,19]. Hence, ZnO NWs could be an attractive material for tissue engineering to prevent infections after scaffold implantation. Therefore, this study proposes and investigates the incorporation of ZnO NWs into PCL scaffolds for tissue engineering applications.

The microstructure of tissue engineering scaffolds has a high impact on cell infiltration, differentiation and cell–cell contact [20], and significant research has been devoted to fabricating ZnO nanomaterial-containing polymers. Among these, electrospinning has gained a great deal of attention owing to its unique and versatile ability to modulate the relationship between structure and performance at the nano-bio interface [21]. For example, ZnO nanoparticles were added to poly (vinylidene fluoride-trifluoroethylene) using electrospinning to improve the sensitivity of the pressure sensor [22]. In addition, due to the high porosity on the sub-micrometre length scale and the high surface area to volume ratio, a lot of ZnO nanostructures modified PCL fibrous scaffolds have been prepared using electrospinning to optimise corrosion resistance and wettability [23] or promote wound healing [24], osteogenesis and angiogenesis [25].

In our previous work, we optimised the synthetic method [19] and evaluated the antibacterial properties of ZnO NWs against *Escherichia coli* (*E. coli*) and *Staphylococcus aureus* (*S. aureus*) [18]; the potential environmental and human health risks associated with ZnO NWs was also assessed [26]. Therefore, in this study, we fabricated a PCL containing ZnO NWs (PCL/ZnO) scaffold using electrospinning for the first time, and evaluated its antibacterial ability against *S. aureus* using the direct contact method. Biocompatibility was detected using a co-culture of the scaffold with HaCaT cells, where cell metabolic activity was investigated. In addition, PCL/ZnO scaffolds can be integrated with various natural or synthetic polymers and ceramic materials for use in bone, cartilage, skin, dental and nerve tissue engineering applications. This study not only lays a foundation for in-depth research on ZnO NWs contained scaffolds but also provides a new view on tissue regeneration engineering.

## 2. Results and Discussion

### 2.1. Preparation and Characterisation of PCL/ZnO Scaffolds

#### 2.1.1. Fabrication and Characterisation of ZnO NWs

**Figure 1:**
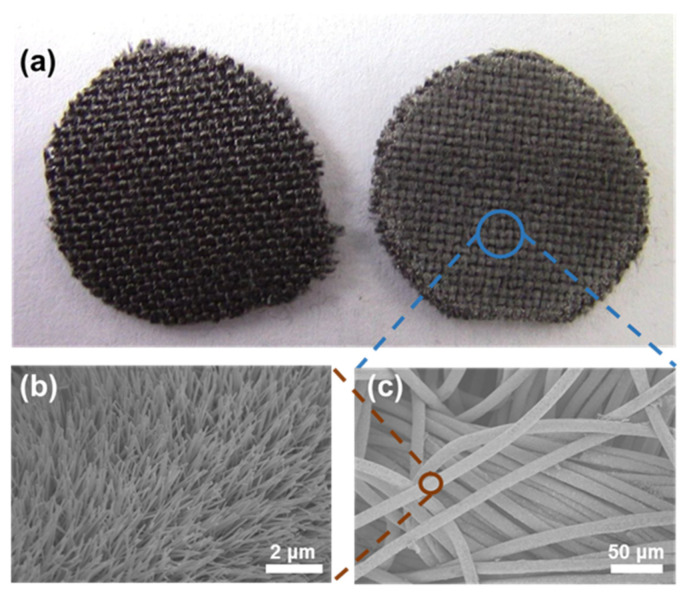
Fabrication and characterisation of the ZnO NWs. (**a**) Carbon cloth with a diameter of 10 mm before (left) and after (right) ZnO NWs growth. (**b**) ZnO NWs grown on one fibre of carbon cloth imaged using SEM. (**c**) ZnO NWs grown along the textile fibres of carbon cloth.

In this study, ZnO NWs were prepared using a wet-chemical method, as previously reported [19]. The soft textile of the carbon cloth was selected and washed in preparation for the growth of the ZnO NWs. Figure 1a shows the overall view of the carbon cloth with a diameter of 10 mm before (left) and after (right) ZnO NWs growth. Figure 1b shows a scanning electron microscopy (SEM) image of the ZnO NWs grown conformably along each textile fibre of the carbon cloth; the diameter of the fibre was approximately 15 μm. The ZnO NWs were pointing outwards without any aggregating, as shown in the magnified SEM image (Figure 1b), and the length of the ZnO NWs was about 3.5 μm. The diameters of the ZnO NWs were in the range of 10 to 40 nm.

#### 2.1.2. Electrospinning of PCL/ZnO Scaffolds

**Scheme 1:**
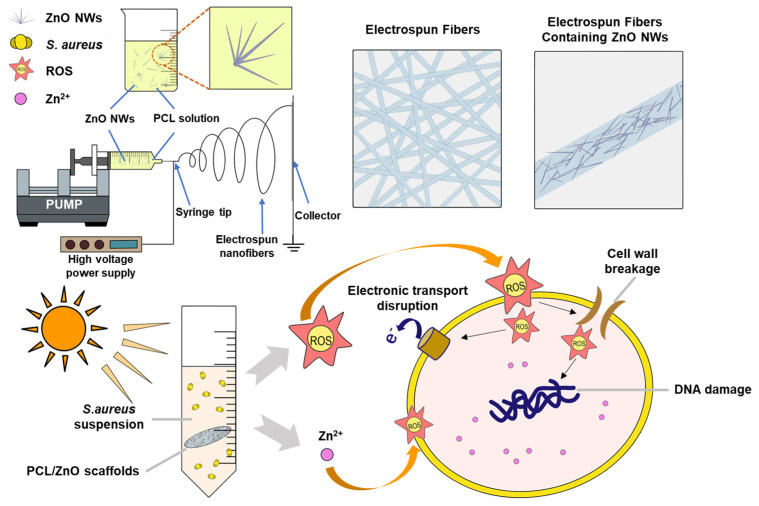
Schematic diagram of the preparing of PCL/ZnO scaffolds using electrospinning and the underlying antimicrobial mechanism.

**Figure 2:**
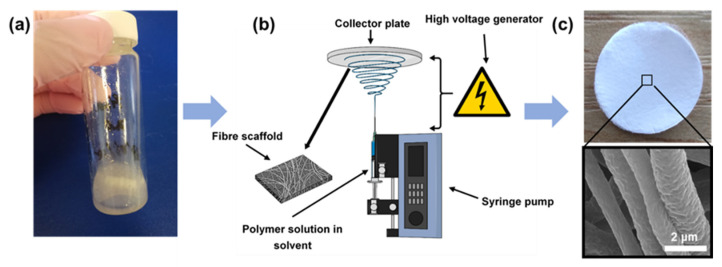
Preparation of electrospun PCL/ZnO scaffolds. (**a**) The ZnO NWs were dissolved in PCL suspension to obtain a thorough mixture. (**b**) Experimental setup for electrospinning. (**c**) Electrospun PCL/ZnO nanofiber membranes and magnified SEM image of the fibres.

The electrospinning solution was prepared by mixing ZnO NWs and a PCL suspension. As electrospinning requires polymer solutions or melts to form polymer fibres, so it can be used to prepare thorough mixtures of polymers with other materials, such as ZnO nanofibers [27] or other nanostructures [28], and then to produce uniformly electrospun fibres. Therefore, the ZnO NWs were firstly acquired using a dip ZnO NWs/textile hybrid in ethanol and subjected to ultrasonic shaking for 10 min, then the ZnO NWs were dislodged and dissolved in ethanol solution. The mixture was then placed in an oven at 50 °C overnight to ensure the complete evaporation of ethanol. Finally, the ZnO NWs were weighed and added to the PCL suspension, as shown in Figure 2a and Scheme 1. The ZnO NWs and PCL suspensions were mixed thoroughly in an ultrasonic bath and stirred for 30 min. Then, the mixed suspension was pipetted inside a 1 mL syringe and propelled under an electric field of 17 kV to ensure that the polymer fibres were formed as the solvent evaporated. A collector at a distance of 20 cm was used to collect the fibres from the PCL/ZnO suspension (Figure 2b). Figure 2c shows the optical and SEM images of the PCL/ZnO scaffold and fibres, showing that the fibres were distributed uniformly with a diameter of approximately 2 μm.

#### 2.1.3. Characterisation of PCL/ZnO Scaffolds

**Figure 3:**
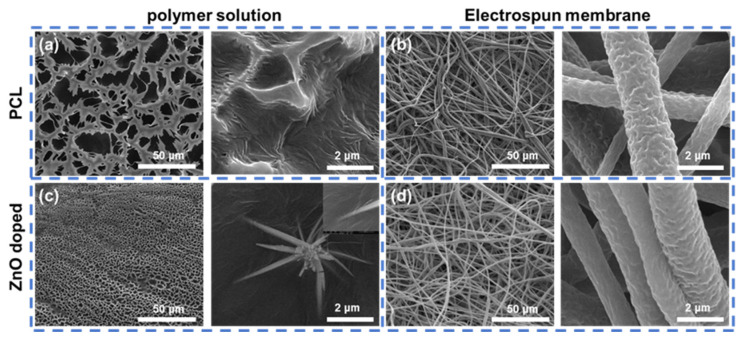
Characterisation of the electrospinning suspension and PCL/ZnO scaffolds using SEM. (**a**) SEM image of the PCL polymer suspension. (**b**) SEM image of the electrospun PCL membrane. (**c**) SEM image of PCL polymer suspension containing ZnO NWs. (**d**) The SEM image of the PCL/ZnO scaffolds.

We know that the agglomeration of ZnO nanomaterials due to the high surface area is still a challenge that has an adverse effect on the antimicrobial property or biocompatibility [29]. To ensure that the ZnO NWs were incorporated into the PCL suspension and to establish whether the ZnO NWs were agglomerated, SEM images of the polymer solution before and after the addition of ZnO NWs were obtained. From Figure 3a,c, we found that ZnO NWs were present and dispersed in the PCL solution without serious damage or breakage. In addition, we can also conclude that the porosity was finer in the ZnO NWs containing PCL suspension. The average pore sizes of PCL and PCL/ZnO suspension were 12.11 ± 0.96 μm and 2.17 ± 0.31 μm. This phenomenon may be because the ZnO NWs filled the interspace of the PCL suspension or because of the increased conductivity caused by ZnO NWs incorporation [30].

In addition to the electrospinning suspension, the electrospun scaffolds with or without ZnO NWs incorporation were also characterised using SEM to detect the morphology features, and it was found that the incorporation of ZnO NWs in PCL had a negligible impact on the morphology of the PCL fibres. The fibres were approximately 2 μm in diameter and randomly and evenly dispersed in the electrospun PCL membrane (Figure 3b,d). This demonstrates that the incorporation of ZnO NWs was superior to that of ZnO nanoparticles because the nanoparticles are easy to agglomerate and could make the surface of the electrospun fibres slightly rough and uneven [31].

**Figure 4:**
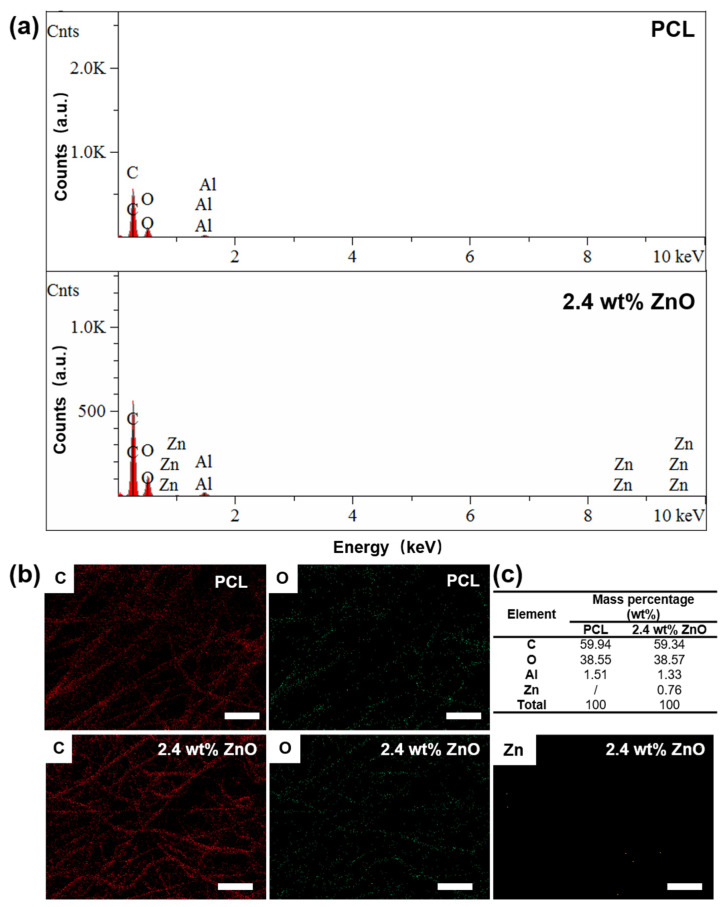
EDX Characterisation of PCL/ZnO scaffold. (**a**) The EDX spectrum image of PCL and 2.4% wt% PCL/ZnO scaffolds. (**b**) C (red), O (green) and Zn element (orange) analysis of PCL and 2.4% wt% PCL/ZnO scaffolds using EDX. (**c**) The mass percentage of PCL and 2.4% wt% PCL/ZnO scaffolds measured using EDX. Scar bar is 10 μm.

After the characterisation of morphology, the element analysis and the presence of ZnO NWs in the PCL/ZnO scaffold were further confirmed using Energy-dispersive X-ray spectroscopy (EDX). As shown in Figure 4, the EDX spectrum and element distribution of PCL and 2.4 wt% PCL/ZnO scaffolds illustrated the presence of Zn element in the PCL/ZnO scaffold. The mass percentage of Zn element was calculated as 0.76 wt% in 2.4 wt% PCL/ZnO scaffolds, which is equivalent to 0.95 wt% ZnO NWs content. The amount of ZnO NWs present in the PCL/ZnO scaffold was not comparable to the ZnO content in the polymer solution before electrospinning. This may be because the element content cannot be accurately determined using in situ EDX analysis since the amount of Zn element was relatively low. Furthermore, the element content analysis using EDX was selected area analysis, which cannot represent the whole sample completely. Moreover, we also found Al element in the EDX analysis results, which was not present in our electrospun PCL scaffold samples. It may be because the sample holder was an aluminium product, and hence the sample holder signal was acquired due to the thinness of the PCL scaffold samples.

### 2.2. Antimicrobial Ability of PCL/ZnO Scaffolds

#### 2.2.1. The Dose of ZnO NWs on Antimicrobial Ability

**Figure 5:**
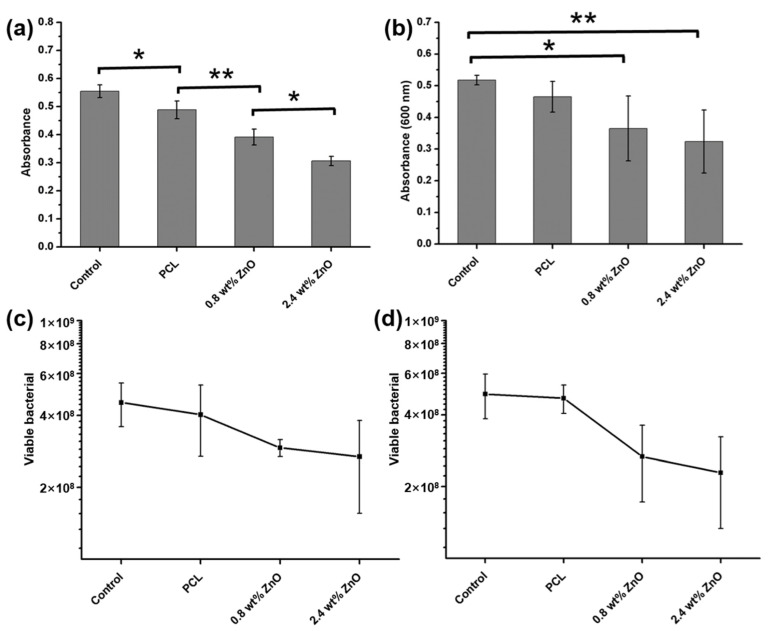
ZnO NWs dose-dependently sterilised PCL/ZnO scaffolds against *S. aureus*. (**a**) Absorbance of bacterial suspension at 600 nm after co-culture with various scaffolds for 16 h. (**b**) Absorbance of bacterial suspension after co-culture with various scaffolds for 24 h. (**c**) Viable bacteria in the bacterial suspension after co-culture with various scaffolds for 16 h detected using the colony counting method. (**d**) Viable bacteria in the bacterial suspension after co-culture with various scaffolds for 24 h detected using the colony counting method. * *p* < 0.05, ** *p* < 0.01.

**Figure 6:**
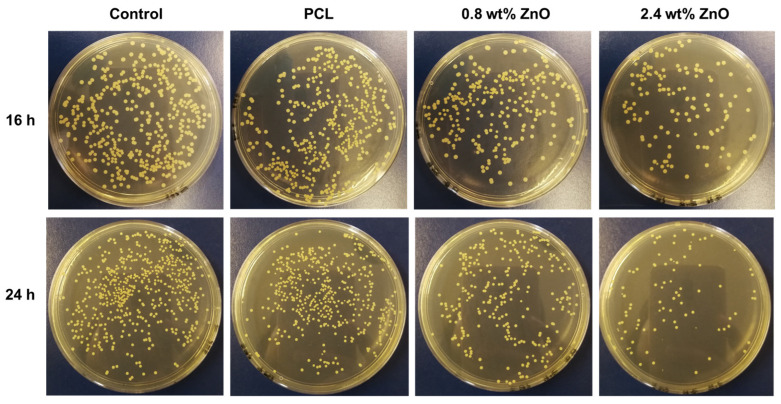
Bacterial colonies after *S. aureus* came directly into contact with PCL, 0.8 wt% and 2.4 wt% PCL/ZnO scaffolds for 16 and 24 h, respectively.

After morphological and element characterisation of the electrospun PCL/ZnO scaffolds, their antimicrobial properties were determined using the direct contact method. A common pathogenic bacterium, *S. aureus*, was used, as shown in Figure 5a. After the *S. aureus* suspension was co-cultured with PCL and PCL/ZnO scaffolds for 16 h, the absorbance of the bacterial suspension was detected. In a control group without a scaffold, the *S. aureus* suspension cultured in PBS for 16 h demonstrated an OD value of 0.555 ± 0.023 at 600 nm, whereas the values were 0.488 ± 0.032, 0.391 ± 0.028 and 0.307 ± 0.017 in the PCL scaffold, 0.8 wt% and 2.4 wt% ZnO NWs contained PCL scaffold (0.8 wt% and 2.4 wt% PCL/ZnO scaffolds) groups. There were statistical differences between the PCL and PCL/ZnO scaffolds, which indicated that the antibacterial property of the scaffold was enhanced with the addition of ZnO NWs and further enhanced with the increased amount of ZnO NWs. This increasing trend was further verified using the colony counting method, as shown in Figure 5c and Figure 6; with the addition of 0.8 wt% ZnO NWs, the number of viable bacteria decreased from 4.03 × 10^8^ to 2.93 × 10^8^, and the amount of viable bacteria further decreased to 2.69 × 10^8^ when the added ZnO NWs were increased to 2.4 wt%. That is to say, compared to the PCL scaffold, the corresponding sterilization rates of 0.8 wt% and 2.4 wt% PCL/ZnO scaffolds were 27.3% and 33.3%. As previously reported, the antibacterial activity of ZnO NWs was caused by the production of reactive oxygen species (ROS) and the released Zn^2+^ from ZnO NWs [18]. ROS can attack the cell wall and lead to cell wall breakage, resulting in the death of bacteria [32,33]. When Zn^2+^ comes into contact with bacteria, Zn^2+^ is absorbed by the bacterial membrane and affects the stability and permeability of the membrane [34]. On the other hand, more Zn^2+^ accumulation will allow the entry of Zn^2+^ into the membrane, and Zn^2+^ ions will interact with nucleic acids and disturb bacterial respiration [35]. Therefore, the amount of ROS and Zn^2+^ increased with the addition of ZnO NWs, leading to an increased sterilization rate with the amount of added ZnO NWs.

#### 2.2.2. The Effect of Disinfecting Time on Antimicrobial Ability

After that, the effect of disinfecting time on antibacterial property was detected by prolonging the contact time to 24 h. The *S. aureus* suspension without scaffold showed an OD value of 0.518 ± 0.015, while the PCL scaffold, 0.8 wt% and 2.4 wt% PCL/ZnO scaffolds showed OD values of 0.465 ± 0.049, 0.365 ± 0.103 and 0.324 ± 0.099, as shown in Figure 5b. There was a statistical difference between the control (*S. aureus* suspension only) and PCL/ZnO scaffold groups but there was no statistical difference between PCL and PCL/ZnO scaffolds, which may be caused by the individual differences in groups because the standard deviation of the three scaffold groups was higher than that of the 16 h contact groups. Hence, the colony counting method was used to explore the sterilization rate of the PCL/ZnO scaffolds deeply. As shown in Figure 5d and Figure 6, the viable bacteria were decreased from 4.70 × 10^8^ to 2.67 × 10^8^ and 2.29 × 10^8^, with the addition amount of ZnO NWs from 0% to 0.8 wt% and 2.4 wt%. In another words, the sterilization rates of 0.8 wt% and 2.4 wt% PCL/ZnO scaffolds were 43.2% and 51.3%, respectively, compared to the simple PCL scaffold group, demonstrating that the sterilization rate was enhanced as the contact time increased from 16 h to 24 h. These results may be attributed to the increased production of ROS and the increased dissolution of Zn^2+^ with contact time [36]. Moreover, the contact duration of ROS and Zn^2+^ with bacteria also contributes to the antibacterial efficiency.

#### 2.2.3. The Effect of Visible Light on Antimicrobial Ability

**Figure 7:**
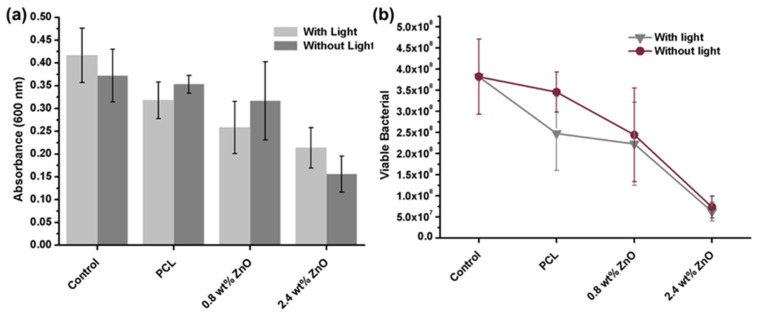
Effect of visible-light irradiation on the antibacterial properties of PCL/ZnO scaffolds. (**a**) Absorbance of bacterial suspension with/without visible light irradiation after co-culture with various scaffolds for 40 h. (**b**) Viable bacteria in suspension with/without visible light irradiation after co-culture with various scaffolds for 40 h detected using the colony counting method.

ZnO is a wide-band-gap semiconductor material and has been used in the photocatalytic degradation of organic pollutants under UV or visible light irradiation, and its antibacterial properties can also be excited by UV or visible light [37]. The normal laboratory environment has fluorescent lighting and can emit UV light; thus, visible light in the laboratory may influence Zn^2+^ release and ROS production from scaffolds [38]. Hence, the effect of visible light on the antibacterial properties of the PCL/ZnO scaffolds was investigated. As shown in Figure 7a,b, regardless of the absorbance of the bacterial suspension or the amount of viable bacteria detected using colony counting, there was no significant difference in darkness and visible light irradiation groups after 40 h of contact with PCL/ZnO scaffolds. This may be because ZnO has a wide-band-gap energy of 3.2 eV, and the UV light over a certain amount could active ZnO NWs to produce ROS [33,39]. However, this photocatalytic efficiency is limited owing to the high recombination rate of photogenerated electrons and holes. Furthermore, fluorescent lighting in a laboratory environment can emit ≤ 4% UV light [37]. Therefore, visible light irradiation and/or the amount of added ZnO NWs may be too low to cause an antibacterial ability change in our designed PCL/ZnO scaffolds. In addition, Nguyen et al. also found no change in antibacterial action of nano-ZnO (at concentrations of 8, 16, and 40 mg/mL) against *E. coli* under visible light irradiation using the agar-well diffusion method [40].

### 2.3. The Biocompatibility of PCL/ZnO Scaffolds

**Figure 8:**
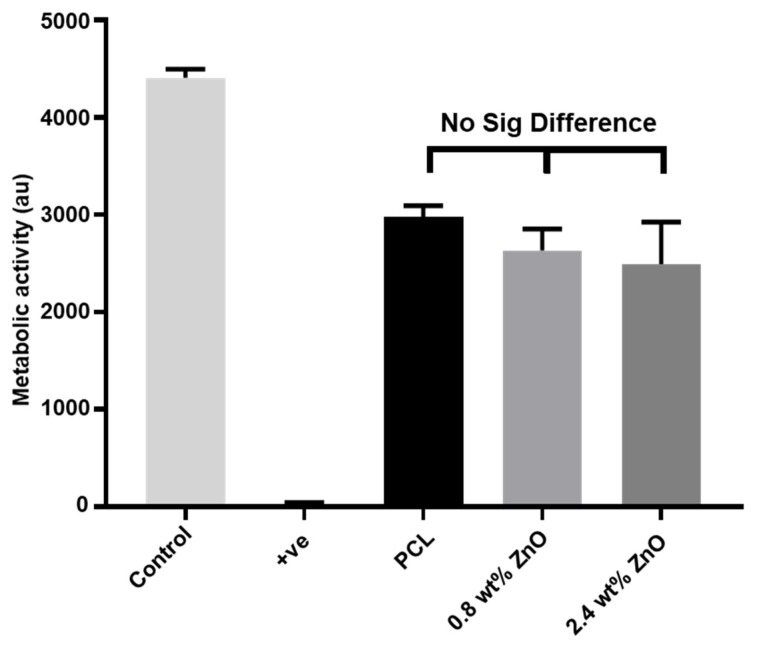
The metabolic activity of the PCL/ZnO scaffold measured using PrestoBlueTM assay after co-culture with HaCaT cells for 3 days.

In addition to the antibacterial property, the biocompatibility of the PCL/ZnO scaffolds was measured by cell metabolic activity using the PrestoBlueTM assay. HaCaT cells of the keratinocyte cell line (specialised skin cells) were used, and HaCaT cells with DMSO served as a negative (cytotoxic) control. As shown in Figure 8, all scaffold groups were significantly different from the negative control group and cell-only positive control, but no statistical difference was found between the three scaffold groups. The difference between the scaffolds and cell-only group may be caused by cell loss during the scaffold transfer process, since we transferred the scaffolds to a new well plate 24 h after cell seeding to avoid the influence of cells growing on the well plate. However, there were no statistical differences among the three scaffold groups, illustrating that ZnO NWs doping had no adverse impact on cell metabolic activity. Furthermore, PCL polymers have been recognised and approved by the FDA for numerous biomedical applications [41], and the addition of ZnO NWs has no adverse effect on cell metabolic activity, demonstrating that the fabricated PCL/ZnO scaffold has good biocompatibility and can be used in tissue engineering.

## 3. Materials and Methods

**Preparation of ZnO nanowires**. The ZnO NWs were grown on carbon cloth using a wet-chemical method, as previously reported [18]. In brief, the carbon cloth was firstly cleaned with acetone and ethanol three times, and then treated with oxygen plasma using a gas plasma cleaner for 5 min to improve hydrophilicity. Subsequently, the ZnO NWs seed solution was pipetted onto a carbon cloth to initiate the ZnO NWs growth. ZnO NWs were grown in an oven at 95 °C for 4 h. Then, the acquired ZnO NWs grown on carbon cloth were taken out from oven and washed three times using distilled water. After that, the carbon cloth was put in an oven at 60 °C overnight for drying. Then, the dried ZnO NWs contained in the carbon cloth were placed in an ethanol solution and ultrasonically shaken for 10 min to dissolve the ZnO NWs. Subsequently, the ethanol solution containing the ZnO NWs was placed in an oven at 50 °C overnight to accelerate the evaporation of ethanol.

**Electrospun polycaprolactone (PCL) and incorporation of ZnO NWs**. The fabrication method for the PCL suspension was reported in our previous work [42]. Briefly, a solvent mixture was prepared using 90% dichloromethane (DCM, Sigma Aldrich, Gillingham, UK) and 10% dimethylformamide (DMF, Sigma Aldrich, Merck Life Sciences, Darmstadt, Germany) and stirred for 2 h to ensure complete mixing. Then, 1.5 g PCL (average 80,000 Mn, Sigma, Merck Life Sciences) was dissolved in the solvent mixture to form 10 wt% PCL. Finally, the solvent mixture was added to the evaporated ZnO NWs at 0.8 wt% and 2.4 wt% content of the PCL suspension. The PCL/ZnO NWs mixture was then placed in an ultrasonic bath for 30 min and stirred for another 30 min to obtain an even solution. Then, a 1 mL Luer-Lok syringe (BD, EU) was used to pipette the PCL/ZnO NWs mixtures, which were used for electrospinning. The mixtures were propelled at a speed of 1 mL/h using an electric field of 17 kV onto a collector, where polymer fibres were formed as the solvent evaporated, and the spinning distance between the needle and collector was 20 cm.

**SEM characterisation**. The electrospun scaffold was imaged using a TESCAN VEGA3 SEM (Tescan, Brno, Czech Republic), and the accelerating voltage was set to 15 kV. The gold coating was sputtered onto the scaffold prior to observation. For the polymer solution, 100 μL was pipetted and dropped onto the cleaning silicon chip, dried in an oven overnight, and sputtered with a gold coating for SEM image acquisition.

**EDX characterisation**. The scaffolds were imaged and analysed using a filed-emission scanning electron microscopy energy dispersive X-ray spectroscopy (FESEM-EDX, Hitachi SU8020) at a voltage of 20 kV. The scaffolds were adhered to an Al sample holder by conductive adhesive, then the gold coating was sputtered onto the scaffolds using an ion sputter coater before observation.

**Bacterium cultivation**. The bacterium used in this study was *Staphylococcus aureus* (*S. aureus*), which is the most common cause of infection. *S. aureus* was activated by being cultured in a brain–heart infusion medium at 37 °C overnight in a shaker with 120 r/min. Then, logarithmic phase bacteria were acquired by culturing the activated *S. aureus* in fresh medium for another 6 h. The bacterial suspension was centrifuged and resuspended in PBS for antimicrobial detection. The final bacterial concentration was adjusted to 10^8^ CFU/mL.

**Antimicrobial properties of the PCL/ZnO scaffolds**. The electrospun PCL/ZnO scaffolds were cut into a round with a diameter of 10 mm using a cork borer, and then the scaffolds were placed into a tube containing 1 mL bacterial suspension. After shaking for 16 and 24 h, the absorbance of the bacterial suspension was measured at 600 nm. Additionally, at the same time point, viable bacteria in suspension were evaluated using the colony counting method. Briefly, the bacterial suspension was collected and ten-fold diluted afterwards, and 200 μL was pipetted and plated onto brain–heart infusion agar plates. The bacteria-containing agar plates were placed and cultured for 24 h at 37 °C, after which CFU counting was carried out to calculate the sterilization rate.

**Biocompatibility of the PCL/ZnO scaffold**. As mentioned above, the electrospun scaffolds were cut into round pieces with a diameter of 10 mm using a cork borer. The scaffolds were sterilized by soaking in 75% ethanol for 30 min. The scaffolds were then washed with distilled water for 5 min three times before the biocompatibility measurement. The direct contact method was used to investigate the toxicity of the scaffolds, and HaCaT cells were used in this study. The scaffolds were put into a 24-well plate, and 1 mL Dulbecco’s modified Eagle’s medium (DMEM) was added to the corresponding wells for 30 min. Next, 1 mL of HaCat cells at a concentration of 30,000 cells/mL was added to each well. After incubation with the scaffold for 24 h, the scaffolds were transferred to a new well plate to avoid the influence of cells growing on the well plate rather than the scaffolds. The cell metabolic activity was investigated by adding PrestoBlueTM to the HaCaT cells that co-cultured with different scaffolds. Six replicates were performed for all groups.

## 4. Conclusions

A ZnO NWs-doped electrospun PCL scaffold was developed using electrospinning for the first time. It is non-toxic to HaCaT cells and is capable of inhibiting the growth of *S. aureus*, achieving a sterilization rate of 51.3%. SEM images and EDX analysis showed that ZnO NWs were successfully doped into the PCL electrospun fibres, and the ZnO NWs significantly improved the antibacterial property against *S. aureus* with the antimicrobial effect of the 2.4 wt% PCL/ZnO scaffold calculated at a 33.3% increase compared with the PCL only scaffold after direct contact for 16 h. Furthermore, this inhibition increased with disinfecting time, the sterilization rate increasing to 51.3% after contact for 24 h. In summary, this PCL/ZnO scaffold was non-toxic to mammalian cells and exhibits certain antibacterial activity, which shows its potential application in tissue engineering to meet clinical needs.

## Data Availability

There are no new data created.
